# Rest and Dobutamine stress echocardiography in the evaluation of mid-term results of mitral valve repair in Barlow's disease

**DOI:** 10.1186/1476-7120-5-17

**Published:** 2007-03-26

**Authors:** Giovanni Minardi, Carla Manzara, Giovanni Pulignano, Giampaolo Luzi, Daniele Maselli, Giovanni Casali, Francesco Musumeci

**Affiliations:** 1 Division of Cardiology, Department of Cardiology and Cardiovascular Surgery, Azienda Ospedaliera San Camillo-Forlanini, Rome, Italy; 2 Division of Cardiovascular Surgery, Department of Cardiology and Cardiovascular Surgery, Azienda Ospedaliera San Camillo-Forlanini, Rome, Italy

## Abstract

**Background:**

Surgical "anatomical" repair is the most frequent technique used to correct mitral regurgitation due to severe myxomatous valve disease. Debate, however, persists on the efficacy of this technique, as well as on the durability of the repaired valve, and on its functioning and hemodynamics under stress conditions. Thus, a basal and Dobutamine echocardiographic (DSE) study was carried out to evaluate these parameters at mid-term follow-up.

**Methods and Results:**

Twenty patients selected for the study (12 men and 8 women, mean age 60 ± 9 years) underwent pre- and post-operative transthoracic echocardiography (TTE) and intra-operative transesophageal echocardiography (TEE). At mid-term follow-up (20 ± 5 months) all patients underwent rest TTE and DSE (3 min. dose increments up to 40 microg/Kg/min protocol). Pre-discharge and one-month TTE showed absence of MR in 11 pts., trivial or mild MR in 9 pts. and normal mitral valve area and gradients. Mid-term TTE showed decrease in left atrial and ventricular dimension, in pulmonary artery pressure (sPAP) and grade of MR. During DSE a significant increase in mitral valve area, maximum and mean gradients, sPAP, heart rate and cardiac output and a decrease in systolic annular diameter and left ventricular volume were found; in 6 pts. a transient left ventricular outflow tract obstruction was observed.

**Conclusion:**

Basal and Dobutamine stress echocardiography proved to be valuable tools for evaluation of mid-term results of mitral valve repair. In our study population, the surgical technique employed had a favourable impact on several cardiac parameters, evaluated by these methods.

## Background

Over the last 30 years many surgical techniques have been developed to correct mitral regurgitation (MR) in degenerative valve disease, to prevent the occurrence of left ventricular outflow tract obstruction (LVOTO) and to ameliorate the function and durability of the repaired mitral valve, particularly in patients with extensive myxomatous degeneration of mitral valve leaflets [1-4]. Transesophageal (TEE) and transthoracic (TTE) Doppler echocardiography are the most useful tools to assess intra- and post-operative results of mitral valve repair, defining morphology and function of the repaired valve (area, mean and peak gradients, annular dynamics, the presence and grade of residual mitral regurgitation, the presence of LVOTO) [5-17] Good functional long-term results using these surgical techniques have been demonstrated [18-21]. Debate, however, persists over the long-term stability of correction of MR due to extensive myxomatous degeneration of both leaflets [18-24] and over the hemodynamics of the repaired valve under stress conditions. Exercise echocardiography, considered a physiological stress test, was used to evaluate the hemodynamic changes and to unmask valve dysfunction under exercise conditions [25,26]. However, informations derived from this test are limited because of the difficulty in obtaining adequate Doppler signals due to respiratory-related artefacts or to increased chest wall motion, or because patients are unable or unwilling to perform an exercise protocol. Dobutamine stress echocardiography (DSE) has been proposed as an effective alternative, offering clear images and optimal Doppler signals [27,28]. However, Dobutamine itself can produce a transient LVOTO, not necessarily related to surgical technique[29].

The purpose of the present study was to analyse mid-term results of mitral valve repair and hemodynamic changes during DSE in patients referred to our Institution with extensive myxomatous degenerative mitral valve with the features of Barlow's disease [30].

## Methods

### Patient Population

Between October 1998 and January 2004, a total of 330 patients underwent mitral valve repair for pure MR at our Institution. Of these, 33 patients (10%) had Barlow's disease [30]. Diagnostic findings obtained preoperatively in all patients by TTE and TEE were: hypertrofied papillary muscles, elongation of the subvalvular apparatus, annular dilatation, bileaflet thickening and redundancy with floppy valve and prolapse, reduction of the coaptation area with displacement of the coaptation above the annular plane. All patients had moderately severe or severe MR. The diagnosis was confirmed at surgery.

Twenty out of the 33 patients, all living in the region, represent the study population.

There were 12 men and 8 women, mean age was 60 ± 9 years (range 54–72 yrs.). At admission 11 patients were in New York Heart Association (NYHA) class II and 9 in class III. All patients underwent basal pre- and post-operative TTE, and, at mid-term follow-up (20 ± 5 months), rest TTE and DSE. All patients underwent pre-operative coronary angiography. All patients gave their informed consent to the study protocol. The research protocol was approved by the locally appointed ethics committee.

### Echocardiographic study protocol

At admission into hospital, all patients underwent TTE and TEE with a HP Sonos 5500 (Agilent Technologies, Andover, MA, USA), using standard protocols (ASE)[31], to evaluate left ventricular diameters (LVEDD, LVESD) and volumes (LVEDV, LVESV), fractional shortening (FS) and Ejection Fraction, Simpson rule (EF), left atrial dimension (LA), pathophysiology of mitral valve prolapse and mitral regurgitation, transmitral diastolic flow, mitral annular diameter, tricuspid regurgitant jet to estimate systolic pulmonary artery pressure (sPAP) by the Bernoulli principle with a fixed right atrial pressure of 10 mmHg.

Intra-operative TEE was performed before cardiopulmonary bypass (CPB) to confirm pre-operative findings and after weaning from CPB to control the competence of the repaired valve, the absence of LVOTO and the adequacy of trans-mitral flow.

TTE was performed before discharge to confirm intra-operative findings and to exclude significant pericardial effusion.

One month later the 20 patients underwent basal TTE; after a mean of 20 ± 5 months follow-up (range 12–44 months), they underwent basal and Dobutamine TTE (3-min dose increments, starting from 5 microg/Kg/min body weight per min and increasing to 10, 20, 30, 40 microg/Kg under continuous electrocardiographic, echocardiographic and blood pressure monitoring). 2D-images and Doppler data were recorded at rest, at each stage, and at 5 min of recovery. The test was discontinued if any of the following end points was met: 1- target heart rate (>85% of maximum predicted); 2- intolerable symptoms (chest pain or progressive dyspnoea); limiting asymptomatic side effects (hypertension defined as >220 mmHg of systolic and/or >120 mmHg diastolic blood pressure; hypotension defined as relative or absolute >30 mmHg decrease blood pressure; supraventricular tachycardia or atrial fibrillation; ventricular tachycardia or frequent, polymorphous premature ventricular beats). The following aspects were evaluated in basal conditions and during stress: the competence of mitral valve, maximum and mean mitral valve gradient by the planimetry of the diastolic CW Doppler velocity signal, the mitral valve area by the pressure half-time formula, the annular dimension from parasternal short-axis view (diastolic and systolic diameter, area and indexed area), sPAP, systolic and diastolic artery blood pressure (PAS, PAD), cardiac output (by the formula: mitral area × velocity time integral of PW diastolic mitral flow × heart rate), LVEDV, LVESV, EF, the possible presence of LVOTO. Measurements were averaged from 5 beats in sinus rhythm or 7 beats in atrial fibrillation. Images of all echo-Doppler examinations were performed by one skilled echocardiologist and stored on video-tape for subsequent analysis.

### Surgical Techniques

The mitral valve was repaired during total normothermic CPB, through conventional midline sternotomy. Myocardial protection was achieved by intermittent anterograde warm blood cardioplegia. The mitral valve was exposed by an incision paralleling Waterston's grove. In presence of ruptured chordae of the posterior mitral leaflet quadrangular resection of the flail segment was performed. In absence of any flail area, part or all the middle scallop of the posterior leaflet was excised. The remnant of the posterior leaflet was detached from the annulus up to the trigons and the height reduced by the excision of its base so as to leave a rim no wider than 1 to 1.5 centimeters. The posterior leaflet was then reattached to the annulus using a 4-0 Ethibond running suture so as to narrow the posterior annulus (sliding technique). Continuity of the posterior leaflet was obtained suturing the two edges with 4-0 Ethibond running sutures. Posterior annuloplasty using a segment of a 3 mm ∅ gore-tex graft completed the repair. The anterior leaflet was approached surgically only in presence of a flail area due to ruptured chordae by inserction of gore-tex cordae passed through the papillary muscles and the free margin of the flail leaflet. One patient with associated CAD underwent myocardial revascularization.

CPB time was 66 ± 25 min. and aortic cross-clamp was 50 ± 19 min.

### Statistical analysis

Continuous variables were expressed as mean ± standard deviation (SD). Differences between continuous variables at baseline and dobutamine stress-echocardiography were evaluated with paired Student's t-test. Discrete variables were summarised by frequency percent and compared with the chi-square test. One-way analysis of variance (ANOVA) for repeated measures, with the Bonferroni correction for multiple testing, was used to measure the variations of the echocardiographic continuous variables measured at baseline, at one month and mid-term follow-up. All analysws were carried out using SPSS 11.0 (SPSS Inc., Chicago, Illinois, USA). A P value of <0.05 was considered as statistically significant.

## Results

Pre-operative TTE and TEE showed in all 20 patients moderately severe or severe MR. Prolapse of posterior leaflet associated or not with ruptured chordae was responsible for MR in all patients. In three patients ruptured chordae to the anterior leaflet were also present. There was mild left ventricular enlargement (LVEDD 61 ± 5 mm, LVEDV 127 ± 45 ml) with preserved systolic ventricular function (EF 60 ± 10%) and mild elevated sPAP (42 ± 16 mmHg). The most important pre-operative TTE findings are listed in Table 1.

**Table 1:** Pre-operative, one month and follow-up echocardiography (20 pts)

**Variable**		**Baseline**	**One month**	**Follow-up**	**p (ANOVA)**
LA size (mm)		50 ± 8	46 ± 6	44 ± 6	ns
LVEDD (mm)		61 ± 5	55 ± 6	52 ± 7	0.01
LVESD (mm)		33 ± 6	35 ± 6	34 ± 9	ns
FS (%)		44 ± 7	36 ± 6	35 ± 10	0.008
LVEDV (ml)		127 ± 4	112 ± 33	114 ± 56	ns
LVESV (ml)		55 ± 30	56 ± 21	57 ± 20	ns
EF (%)		60 ± 10	50 ± 7	54 ± 12	0.038
MR grade	(3+;4+)	11; 9	0;0	ns	0.001*
	(0;1+;2+)	0;0;0	11;8;1	11;8;1	ns**
sPAP (mmHg)		42 ± 16	38 ± 14	30 ± 11	ns

LA = left atrium; LVEDD = left ventricular end diastolic diameter; LVESD = left ventricular end systolic diameter; FS = fractional shortening; LVEDV = left ventricular end diastolic volume

LVESV = left ventricular end systolic volume; EF = ejection fraction

MR = mitral regurgitation; sPAP = systolic pulmonary artery pressure

* baseline vs one month

** one month vs follow-up

Intraoperative TEE, before CPB, confirmed pre-operative findings. After weaning from CPB, all patients were in sinus rhythm. TEE demonstrated optimal leaflet coaptation, normal diastolic transmitral flow, trivial or absent MR, absent LVOTO. The mean post-operative stay was 8.5 ± 2.5 days. No hospital deaths occurred.

At discharge, TTE showed absence of MR in 11 patients (55%) and mild or trivial MR in 9 patients (45%), mean LVEF 50 ± 7%.

One-month TTE showed, in comparison with pre-operative data, a decrease in LA diameter, LV diameter, volume, FS, EF, as well as in grade of MR; no change was found in grade of MR in comparison with discharge results (Table 1).

At the latest follow-up (12 to 44 months after surgery, mean 20 ± 5 months), all patients were hemodinamically stable, 16 patients in NYHA class I and 4 in class II.

TTE findings remained unchanged, with the exception of sPAP, which was significantly reduced to pre-operative values (42 ± 16 vs 30 ± 11 mmHg) (Table 1).

**Figure 1:**
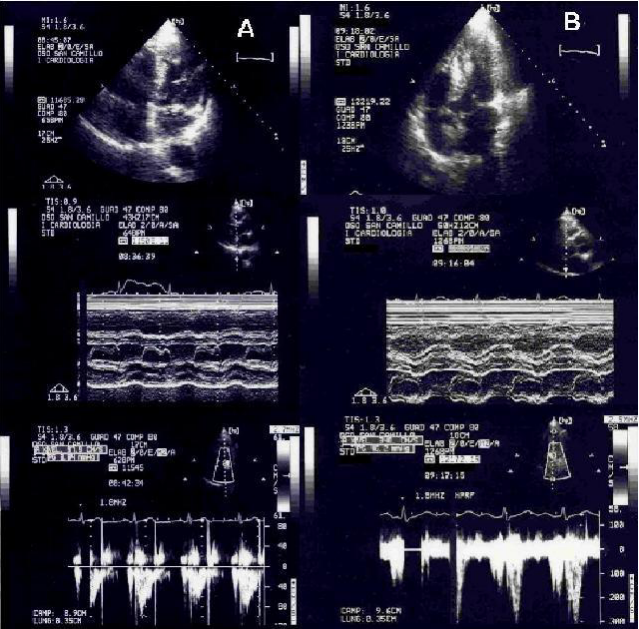
**Rest and Dobutamine echocardiogram following mitral valve repair**. Panel A: Top: Two-dimensional echo, apical 4-chamber view: normal coaptation of mitral leaflets; systolic anterior movement (SAM) is absent. Middle: M-Mode echo, mitral valve: normal coaptation of mitral leaflets. Bottom: Two-dimensional echo, PW Doppler in left outflow tract (LVOT):normal peak systolic velocity. Panel B: Top: Two-dimensional echo, apical 4-chamber view: SAM of anterior mitral leaflet is present. Middle: M-Mode echo, mitral valve; SAM is present. Bottom: Two-dimensional echo, PW Doppler in LVOT: elevated peak mid-systolic velocity corresponding to 46 mmHg outflow gradient.

During DSE mitral valve area increased significantly, as well as maximum and mean gradients (p < 0.001), sPAP, HR, CO, whereas systolic annular diameter, LVEDV, LVESV decreased significantly. No significant difference was found among other echocardiographic parameters (Table 2). At peak stress six patients showed a transient LVOTO, with a peak gradient <40 mmHg in two and >40 mmHg in four (>100 mmHg in two patients) (Figure 1); one patient showed trivial MR, which was absent at rest. There were no stress-induced wall motion abnormalities.

**Table 2:** Baseline and dobutamine stress echocardiography (20 pts)

**Variable**	**Baseline**	**Stress**	**P**
Mitral valve area (cm^2^)	2.9 ± 0.7	4.5 ± 2	<0.001
Mx PG (mmHg)	7.2 ± 1.9	14 ± 3.7	0.4
Md PG (mmHg)	2.7 ± 1	6.3 ± 1.5	<0.001
Mitral Annulus, systole (mm)	41 ± 3.4	38 ± 2.2	<0.001
Mitral Annulus, diastole (mm)	43.7 ± 3.3	45.7 ± 3	0.4
Mitral Annulus, area (cm^2^)	3.4 ± 0.4	3.7 ± 0.6	0.1
IAA	1.9 ± 0.3	2.1 ± 0.3	0.1
sPAP (mmHg)	33.5 ± 10	41.6 ± 12	0.03
MR grade (1+/2+)	9/20	10/20	ns
HR (beats/min)	75 ± 19	133 ± 22	<0.001
SBP (mmHg)	124 ± 14	127 ± 24	0.6
DBP (mmHg)	76 ± 9	70 ± 9	0.1
CO (L/min)	3.9 ± 1.9	6.6 ± 2.8	<0.001
EF (%)	50 ± 11	52 ± 12	0.3
LVEDV (ml)	114 ± 58	103 ± 56	0.05
LVESV (ml)	61 ± 50	54 ± 48	0.04
LVOTO (%)	0	30	<0.001

Mx PG: maximum pressure gradient; Md PG: mean pressure gradient; IAA: indexed annular area; sPAP: systolic pulmonary artery pressure; MR: mitral regurgitation; HR: heart rate; SBP: systolic blood pressure; DBP: diastolic blood pressure; CO: cardiac output; EF: ejection fraction; LVEDV: left ventricular end diastolic volume; LVESV: left ventricular end systolic volume; LVOTO: patients with left ventricular outflow tract obstruction.

## Discussion

Many surgical procedures have been developed over the years to correct valve regurgitation in degenerative mitral valve disease, to extend feasibility of the repair to the most complex lesions and to optimize durability [1-4]. Good results in terms of event-free survival (thromboembolism, anticoagulant-related haemorrage, endocarditis), mitral valve function at rest, grade of residual regurgitation and long-term stability of the repair have been reported [18-24]. Nevertheless, some potential problems secondary to the most commonly used surgical technique have been described. LVOTO is caused by systolic anterior motion of the anterior mitral leaflet and may occur in presence of excess tissue of the mitral leaflets and inadequate ring sizing, resulting in a "too small ring for a too large anterior leaflet" [2]. Impaired diastolic transmitral flow, with significant increase in transvalvular pressure gradient and in sPAP at peak exercise, have recently been reported in patients treated by the "double-orifice" technique [25]. Impaired diastolic annular dynamics and hampered exercise-related increase in left ventricular ejection fraction can occur when a rigid ring is employed [32,33]. Calcification of the pericardium, when utilized for the posterior annuloplasty is a possible event [34,35].

TTE and TEE are the most usual and useful tools to evaluate intra- and post-operative results [5-17], while exercise TTE is usually employed to evaluate valvular dynamics and function under stress conditions [25,26].

Our study enrolled 20 patients with Barlow's disease and significant MR. All patients underwent surgical "anatomical" repair, which was supported by posterior annuloplasty, with a segment of a 3 mm ∅ gore-tex tube. This technique has the purpose of preserving the morphological, non planar configuration of the mitral annulus, maintaining its physiology, respecting anatomical and functional valvular-ventricular interaction and preventing the occurrence of LVOTO.

Our results confirmed the efficacy, the stability and durability of mitral valve repair using this technique, as demonstrated by the intra-operative assessment and the one-month and mid-term echocardiographic follow-up. Further support to the good surgical result was the significant improvement in functional class which occurred in all patients. Many published series of follow-up data have shown that mitral valve repair is a safe and reproducible technique, that results are stable, with low risk of re-operation, decrease in LVESD and LVESV and improvement of LV function is achieved in most patients (with NYHA functional class I-II). Moreover, all these studies have demonstrated that together with the elimination of valve regurgitation lower complication rates than with mitral valve replacement is achieved [18-21].

In our study DSE was performed with the aim to evaluate haemodynamic response under stress conditions and elicit the possible dysfunction of the repaired mitral valve. During stress, functional mitral valve area and mean transvalvular gradient increased significantly, as well as sPAP, HR and CO. There was also an increase in annular area, annular diastolic diameter and annular area index, which was not significant. Systolic annular dimension, LVEDV and LVESV decreased significantly. These data, because of the small sample, are not conclusive, but clearly demonstrate evidence of good valve function, not only at rest but also during stress, further supported by a rise in mitral valve gradients and sPAP, as consequence of haemodynamic effects of Dobutamine, which did not reach pathologic levels. Previous study on the normal mitral valve physiology clearly demonstrated that exercise induces increase of transmitral flow by an increase in mean diastolic cross-sectional area [36]. Consequently, maintenance or a minimal limitation of the physiologic diastolic motion of the mitral valve annulus is an important component of surgical repair if unrestrictive transvalvular flow has to be maintained during high flow cardiocirculatory conditions. Correlation between incremental dose of Dobutamine and increase in transmitral peak flow velocity has recently been demonstrated [37]. In patients who underwent mitral valve repair, Borghetti demonstrated, using supine bicycle exercise, absence of significant MR and increase in transmitral flow velocities, LVEF and mitral annular dynamics (evaluated by mitral annular motion) during peak exercise condition, with more favourable results in the subgroup of patients who had annuloplasty performed with autologous pericardium [35]. Other studies have been performed in animal models or in computational models to evaluate mitral annular three-dimensional dynamics and transvalvular gradients using myocardial markers and Doppler echocardiography [28]. Though interesting the results, the major limitations of these studies are inherent to the animal or to the laboratory models used which may not be translated to the clinical setting.

With the technique we describe, mitral annular dynamics have been preserved as demonstrated by the mild, but not significant, increase in diastolic and significant decrease in systolic diameters and by the increase of annular area and annular area index.

During DSE only one patient showed trivial MR which was absent at rest and six patients showed transient LVOTO: two patients with intra-ventricular peak gradient < 40 mmHg, two between 40 and 100 mmHg and two > 100 mmHg. The appearance of LVOTO during DSE in non-operated patients was first described in 1992 [38]. A statistical correlation with a high basal EF and fall in systolic blood pressure during stress has been demonstrated [38] as well as a correlation with left ventricular geometry and systolic volume and FS [39]. In a different clinical setting other Authors found an incidence of LVOTO of 7.5% and a correlation with increased myocardial contractility, systolic anterior motion of the anterior leaflet, decreased venous return to the left ventricle, and peculiar characteristic of the left ventricular geometry [29]. In repaired mitral valve the occurrence of basal LVOTO (a 4–6% incidence) has been reported by several Authors [10-14], as a consequence of surgically induced changes, particularly reduction in circumference of mitral annulus, prosthetic ring implant and narrowing of the mitro-aortic angle, which are responsible for an overlapping of the usually distinct two functional compartments: inflow and outflow. Recently, a correlation between LVOTO appearance and a too small circumference of the mitral annulus following valve repair compared to the combined heights of the two leaflets, being the anterior mitral leaflet height a more important factor, has been demonstrated [17]. Jebara and Coll found an incidence of LVOTO very low (2.4%) when the "sliding technique and prosthetic ring annuloplasty", was used [15]. Transient LVOTO has been observed during exercise test, associated with mitral systolic anterior motion and significant intraventricular gradient, in patients with repaired mitral valve [25]. The absence of LVOTO in basal condition in our series supports the need at surgery of reducing the height of the posterior leaflet, particularly in patients with extensive myxomatous degeneration of both leaflets. The induced and transient LVOTO during DSE is correlated to pharmacological hemodynamcs changes.

## Study limitation

A limitation of the present study is the relatively small sample size (only 20 patients); therefore the statistical analysis could have been adversely influenced by inappropriate study sample.

A long-term clinical and echocardiographic follow-up is necessary to confirm the stability of the repaired valve.

## Conclusion

Transthoracic and transesophageal echocardiography have a strategic role in pre-, intra- and post-operative assessment of the mitral valve, particularly in patients with Barlow's disease. In the present study, TTE and DSE proved to be valuable tools for evaluation of mid-term results of "anatomical" repair of MR due to extensive myxomatous degeneration of both leaflets.

The surgical technique employed had a favourable impact on several parameters. The majority of patients showed competent mitral valve or mild-trivial MR, with no pathological modification under stress conditions. Furthermore, remarkable reduction in LA and LV dimension was clearly demonstrated at follow-up.
